# Microfilaria-dependent thoracic pathology associated with eosinophilic and fibrotic polyps in filaria-infected rodents

**DOI:** 10.1186/s13071-020-04428-0

**Published:** 2020-11-07

**Authors:** Frédéric Fercoq, Estelle Remion, Nathaly Vallarino-Lhermitte, Joy Alonso, Lisy Raveendran, Colin Nixon, John Le Quesne, Leo M. Carlin, Coralie Martin

**Affiliations:** 1 Unité Molécules de Communication et Adaptation des Microorganismes (MCAM UMR 7245), Muséum national d’Histoire naturelle, CNRS, P52, 61 rue Buffon, 75005 Paris, France; 2 CRUK Beatson Institute, Garscube Estate, Switchback Road, Bearsden, Glasgow, G61 1BD UK; 3 Leicester Cancer Research Centre, University of Leicester, Leicester, UK; 4 Institute of Cancer Sciences, University of Glasgow, Glasgow, G61 1GH UK

**Keywords:** Filariasis, Microfilaria, Lung, Polyps, Vascularization, Eosinophils

## Abstract

**Background:**

Pulmonary manifestations are regularly reported in both human and animal filariasis. In human filariasis, the main known lung manifestations are the tropical pulmonary eosinophilia syndrome. Its duration and severity are correlated with the presence of microfilariae. *Litomosoides sigmodontis* is a filarial parasite residing in the pleural cavity of rodents. This model is widely used to understand the immune mechanisms that are established during infection and for the screening of therapeutic molecules. Some pulmonary manifestations during the patent phase of infection with *L. sigmodontis* have been described in different rodent hosts more or less permissive to infection.

**Methods:**

Here, the permissive Mongolian gerbil (*Meriones unguiculatus*) was infected with *L. sigmodontis*. Prevalence and density of microfilariae and adult parasites were evaluated. Lungs were analyzed for pathological signatures using immunohistochemistry and 3D imaging techniques (two-photon and light sheet microscopy).

**Results:**

Microfilaremia in gerbils was correlated with parasite load, as amicrofilaremic individuals had fewer parasites in their pleural cavities. Fibrotic polypoid structures were observed on both pleurae of infected gerbils. Polyps were of variable size and developed from the visceral mesothelium over the entire pleura. The larger polyps were vascularized and strongly infiltrated by immune cells such as eosinophils, macrophages or lymphocytes. The formation of these structures was induced by the presence of adult filariae since small and rare polyps were observed before patency, but they were exacerbated by the presence of gravid females and microfilariae.

**Conclusions:**

Altogether, these data emphasize the role of host-specific factors in the pathogenesis of filarial infections.
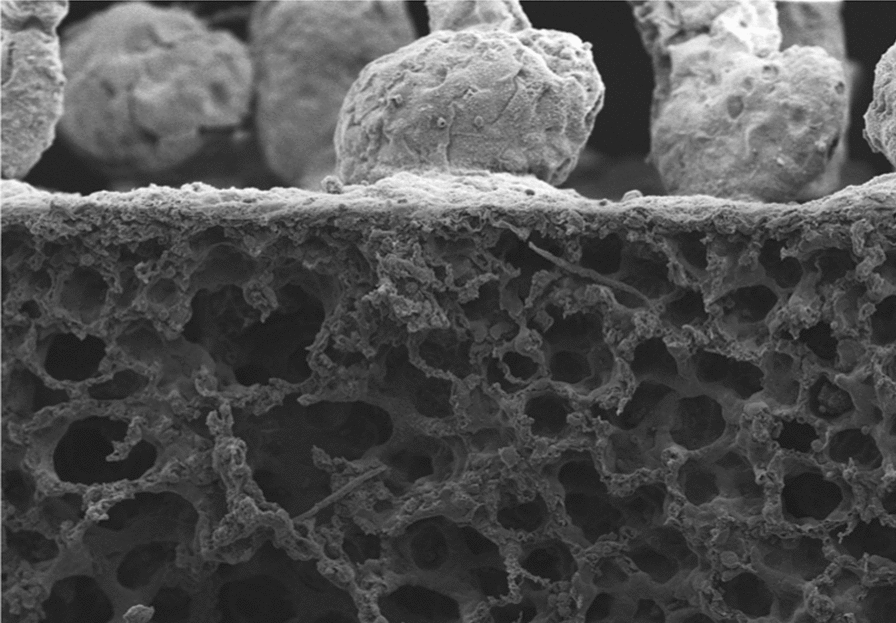

## Background

Many helminth parasites interact with respiratory tissues and the pulmonary immune system. Among them, the filariae *Wuchereria bancrofti*, *Brugia malayi* and *Loa loa* (family Onchocercidae) are major human pathogens [1]. They have evolved a circadian behaviour where the offspring, i.e. microfilariae, cycle between circulating in the blood and sequestering in the pulmonary vasculature [2]. In a small number of individuals infected by *W. bancrofti* and *B. malayi*, immune hyperreaction to microfilariae induce a syndrome called tropical pulmonary eosinophilia [3–5]. In these patients the microfilariae released in blood circulation become trapped in the pulmonary microcirculation and are cleared. The degenerating larvae release antigenic constituents which trigger an immune response that is characteristic of a Type 2 reaction and induce asthma-like symptoms. In addition, the human filaria *Mansonella perstans* has been reported to induce pleural effusion in some clinical cases [6–9]. Finally zoonotic *Dirofilaria immitis* filarial infections often result in pulmonary inflammation and damage due to the migration of larvae and/or adult parasites to the lungs [10–12].

Human filariae are specific to their host. Consequently, the discovery of drugs against filariasis, the study of filarial pathologies or the analysis of the immune response in the vertebrate host rely on substitute filaria. The most commonly used model is the rodent filaria *Litomosoides sigmodontis* which, like *M. perstans,* resides in the serous body cavities, including the pleural spaces [13–15]. The difficulties in breeding and handling of its natural host, the cotton rat *Sigmodon hispidus* gave rise to the search for other adequate laboratory hosts. To date, the gerbil *Meriones unguiculatus*, the murid rodent *Mastomys natalensis*, the albino rat, and the BALB/c mouse have been or are successfully used as experimental hosts of *L. sigmodontis* [16–21]. This filarial species develops a patent infection in these rodents. The infection is cleared in subcutaneous-inoculated mice about 100 days post-infection [22], whereas it can persist for more than a year in gerbils [16, 23]. Microfilariae appear in peripheral blood 8 weeks after infection in about half of infected BALB/c mice and around 9–12 weeks after infection in about 70–90% of infected gerbils [16, 18, 24–26].

Infective larvae migrate from the skin to the pleural cavity [15, 27] where they settle, grow and moult until they reach the adult stage. In the pleural cavity of BALB/c mice, the presence of the filariae induces a granulomatic cell reaction. The first granulomas are observed between day 8 and day 10 and are essentially composed of macrophages and eosinophils. Some contain exuviae of L3 [23]. Later, after the fourth moult, granulomas containing exuviae of young adults present 70% eosinophils but no neutrophils [23]. Finally from day 60 onwards granulomas composed of macrophages, eosinophils and neutrophils contain remains of adult filaria. In parallel to these granulomas, the inflammatory reaction recruits cells to the pleural cavity as early as the L3 arrive there [28, 29]. These infiltrates are mainly composed of macrophages, neutrophils and eosinophils [30] with a peak 30 days post-infection [31]. The proliferation and the alternative activation of these pleural macrophages is promoted by the interleukin (IL)-4 receptor and IL-33 (ST2) in *L. sigmodontis* infected BALB/c mice [32]. The alteration of IL-4/IL-5 signalling alters this cellular environment and leads to increased susceptibility to *L. sigmodontis* infection and influences adult worm burden and/or microfilaremia [24, 33, 34] as well as lung pathogenesis [24, 33]. Particularly, a central role of IL-13 and IL-4 in macrophage activation and eosinophil recruitment and in lung pathogenesis has been highlighted in a recent analysis of cytokine and chemokine transcripts in the lungs of 70 days-infected BALB/c mice [24]. Some lung pathology was also partially described in cotton rats, gerbils and white rats [19, 20, 35] showing a cellular and fibrotic reaction on the outside of the lung pleura. Most of these infected rodents were in various stages of mite-induced infection with *L. sigmodontis* but all animals developed patent infections. However there was no evidence that the findings in these rodents were dependent on the microfilariae. We recently showed that lung pathology in *L. sigmodontis*-infected BALB/c mice is dependent on microfilariae and Th2 immune responses [24]. Here we further describe the pulmonary pleural pathology in *L. sigmodontis-*infected gerbils *Meriones unguiculatus* and discuss the role of microfilariae in the development of lung inflammation. We also highlight the importance of parasite location within the host in the mating process.

## Methods

### Rodents and infestation protocol

The filaria *Litomosoides sigmodontis* Chandler, 1931 is maintained in the National Museum of Natural History (MNHN) facilities and infective third-stage larvae (L3) were recovered by dissection of the mite vector *Ornithonyssus bacoti* as previously described [18, 36].

Eight-week-old gerbils were purchased from Janvier (Le Genest-Saint-Isle, France) and maintained in the MNHN animal facilities on a 12-h light:dark photocycle. Gerbils were inoculated subcutaneously with a single dose of 40 L3 in 200 μl of RPMI1640 (Eurobio, Les Ulis, France) into the left lumbar area.

### Filarial recovery

Gerbils were sacrificed at 70 days post-inoculation (p.i.). Filariae were collected from the pleural cavity, counted, sexed and the presence of microfilariae in female uteri was assessed using light microscopy (Olympus BX63 microscope, DP72 camera, Olympus Europa, Hamburg, Germany).

### Lung imaging by scanning electron microscopy (SEM)

Lungs from *L. sigmodontis* naive (*n* = 3), 50 days-infected (*n* = 3), 70 days-infected microfilaremic (Mf^pos^) gerbils (n = 2) and 70 days-infected amicrofilaremic (Mf^neg^) gerbils (*n* = 1) were removed from the chest, placed in a petri dish containing PBS and cut in 3–4 mm thick sections. Sections were fixed with 2.5% glutaraldehyde for 30 min, dehydrated with increasing concentrations of ethanol (50% for 5 min, 70% for 5 min, 90% for 5 min, 100% for 3 × 5 min), and then with hexamethyldisilane (HMDS) for 2 × 5 min. Thereafter, the samples were immersed in HMDS and were left evaporating overnight under a fume hood. Dried lungs were fixed on metal supports using double-sided carbon tape and metallized by sputtering gold (Jeol FJC-1200 metallizer, JEOL, Peabody, USA). Observations of the visceral pleura were made with a Hitachi SU3500 SEM (MNHN Technical Electron Microscopy Platform, Paris, France; Hitachi, Tokyo, Japan).

### Lung histology and immunohistology

Lungs from naive and *L. sigmodontis* infected gerbils (*n* = 3–19 per group) were inflated with and fixed in 4% buffered formalin overnight. Fixative was changed 24 h post-fixation for a further 24 h. Thereafter, lungs were dehydrated in increasing concentrations of 70% to 100% ethanol baths, and then placed in toluene before paraffin embedding. Four-micron-thick serial sections were prepared. All sections were cut deep enough to see the main bronchi and perivascular adventitial cuff.

Various stainings were performed: (i) Picrosirius red (Bio Optica, Italy) to visualize collagen fibers according to the manufacturerʼs recommendations; (ii) Congo Red staining was performed using the following online protocol http://www.ihcworld.com/_protocols/special_stains/congo_red_highman.htm to visualize eosinophils; (iii) Toluidine blue staing was performed using the following online protocol http://www.ihcworld.com/_protocols/special_stains/toluidine_blue.htm to visualize mast cells; (iv) a cytokeratin immunostaining was used to visualize mesothelial cells: antigen retrieval was performed using a solution of Proteinase K (10 μg/ml) in Tris-EDTA buffer (10 min, 37 °C), then peroxidases and endogenous alkaline phosphatases were blocked by adding Dual Endogenous Enzyme Block for 10min (Dako, Santa Clara, USA). Non-specific sites were blocked with 5% rat-serum in PBS. Sections were incubated with the mouse anti-human cytokeratin monoclonal antibody (Ab) (1/50, clone AE1/AE3, Dako) for 45 min and then rinsed twice with PBS. Binding of the antibodies was detected by HRP linked universal secondary antibody (DAKO) and AEC substrate (DAKO) for 10 min. Sections were then counterstained with a Mayerʼs hematoxylin solution; (v) Immunostaining for leukocytes was performed at the Beatson Institute’s Histology Facility. Antigen retrieval was performed using Leica ER2 retrieval buffer (20 min, 95 °C). Sections were then stained at a previously optimised dilution, CD3: 1/100 (rabbit anti-mouse/human antibody, clone SP7; ab16669, Abcam, Cambridge, UK) and CD45: 1/1000 (rabbit anti-mouse/human antibody, clone ab10558, Abcam) for 30 min at room temperature with the autostainer Bond Rx (Leica Microsystems GmbH, Wetzlar, Germany). Antigen retrieval was performed on histological sections for CD68 (mouse anti-human antibody, clone PG-M1; M0876, Agilent, Santa Clara, USA) using Target Retrieval Solution, high pH (Agilent) (20 min at 97 °C) on a Dako PT retrieval module. CD68 sections were then stained on-board a Dako Autostainer Link48 platform at 1/500. All the IHC sections were visualised with 3,3’Diaminobenzidine.Stained sections were then digitally scanned using a Leica Aperio AT2 slide scanner at ×20 magnification.

Hearts and diaphragms fixed in 4% buffered formalin were also embedded into paraffin and 4-µm-thick sections were prepared. The tissues were deparaffinized with toluene and then hydrated using a series of decreasing concentrations of ethanol and a hematoxylin-eosin staining was performed. Visceral pleura, pericardium and diaphragmatic pleura were analyzed by light microscopy (Olympus BX63 microscope, DP72 camera) using the cell Sens Dimension 1.9 software.

### Tissue processing and immunostaining for 3D imaging

Some paraffin-embedded tissues were used for 3D imaging after histological analysis. The protocol was adapted from [37]. The blocks were first deparaffinized by warming at 60 °C to melt the paraffin and immersing in 3 consecutive baths of xylene (1 h/8 h/1 h). Samples were then rehydrated in decreasing concentrations of 100% to 70% ethanol baths, and placed in PBS. Samples were stored in PBS/Bovine serum albumin (BSA)1%/Azide 0.05% at 4 °C.

For 2-photon imaging, the left lung was isolated and cut with a vibrating microtome (Campden5100mz) into 500-μm slices. The post-caval lobe was kept intact and used for light sheet microscopy imaging.

Samples were permeabilized for 24 h in PBS/Neutral goat serum (NGS) 10%/BSA1%/TritonX-100 (Tx100) 0.3%/Azide 0.05% at 37 °C and stained for 48 h with Cy3 anti-mouse/human αSMA antibody (clone 1A4, Sigma-Aldrich, Taufkirchen, Germany) in PBS/NGS10%/BSA1%/TX-100 0.1%/Azide 0.05% at 37 °C. Samples were then washed for 24 h in PBS/BSA1%/TX-100 0.1%/Azide 0.05% at 37 °C and 2 × 1 h in PBS before and after 4% paraformaldehyde post-fixation.

Samples were dehydrated in increasing concentrations of 50–100% methanol baths and cleared with Ethyl Cinnamate (Sigma Aldrich). 500-μm slices were transferred to a glass slide with slide chambers (Frame-Seal, Biorad, Hercules, USA), covered with Ethyl Cinnamate and a coverslip. Images were acquired with a Zeiss 880 2-photon microscope (Beatson Advanced Imaging Ressources, Beatson Institute, Glasgow, UK; Carl Zeiss, Oberkochen, Germany) equiped with a 32 channel Gallium arsenide phosphide (GaAsP) spectral detector (Carl Zeiss, Oberkochen, Germany) using 20×/1 NA water immersion objective lens. Samples were excited with a tunable laser (680–1300 nm) set up at 950 nm and signal was collected onto a linear array of the 32 GaAsp detectors in lambda mode with a resolution of 8.9 nm over the visible spectrum. Spectral images were then unmixed with Zen software (Carl Zeiss) using references spectra acquired from unstained tissues (tissue autofluorescence and second harmonic generation) or beads labelled with Cy3 anti αSMA antibodies.

Intact lobes were immersed in Ethyl Cinnamate in an imaging chamber and imaged with a M Squared Aurora Light Sheet Microscope (IMPACT facility, Centre for Discovery Brain Sciences, Edinburgh, UK) using 488 nm and 568 nm laser lines.

### Analysis of lung pathology

Histology images were analysed using Sens Dimension 1.9 software. To analyse pleural inflammation, full lobe sections were imaged by mosaic imaging and pleural pathology (100 × length of pathologic pleura / total perimeter) was measured. Only the support area of the polyp on the mesothelium, or pedicle, was measured to evaluate this percentage.

To analyse immune composition of polyps, digital slides were analysed with QuPath [38]. All polyps/subpleural foci from the sections were manually annotated and DAB/Congo red/toluidine blue positive cells within the annotated areas were detected using the “positive cell detection” tool. The stain vectors used for detection were adjusted for each slide to correct any difference in staining/acquisition. Results were then expressed as number of positive cells/mm^2^ of polyp/subpleural foci.

2-photon and light sheet microscopy images were analysed using the IMARIS software v9 (Bitplane, Oxford Instruments, Abingdon, UK). The different planes (z) were stacked to obtain a three-dimensional reconstruction of the different fluorescence signals. The different structures (blood vessels, polyps, bronchi, pleura) were segmented manualy using autofluorescence and alpha-smooth muscle actin (αSMA) signals. Blood vessels segmentation was started by drawing main veins/arteries which were then followed to segment the full vascular tree.

The volume of each segmented polyp and the surface of the pleura were exported and used to calculate the concentration of polyps of different size (No. of polyps/mm^2^ of pleura).

### Statistical analyses

The choice of statistical tests was based on the sample size and on the Shapiro-Wilk test to determine whether the distribution of the samples conformed to a normal distribution. Data from separate experiments were pooled when possible (homoscedascity). The number of parasites was compared between the groups of gerbils (Mfneg *versus* Mfpos). Normally distributed data were analyzed using Student’s t-test.The proportion of pathological pleura was compared between the groups of gerbils (naive *versus* Mfneg *versus* Mfpos). Non-parametrically distributed data were analyzed using the Kruskal-Wallis test followed by Dunn’s multiple comparisons *post-hoc* test for a further comparison of the groups. The correlation between pleural pathology and the presence of filariae (adult male and/or female, or microfilariae) was analyzed using the Spearmanʼs rank correlation test then a linear regression was performed to describe relationship between variables (the pleural pathology is the dependent variable and the presence of filariae is the explanatory variable). Representation and data analyses were performed with R 3.6.1 and Prism 6.0 software (GraphPad Inc.,San Diego, USA). Statistically significant *P*-values are given or indicated as *P < 0.05.

## Results

### Amicrofilaremic gerbils have fewer parasites in their pleural cavity

**Fig. 1:**
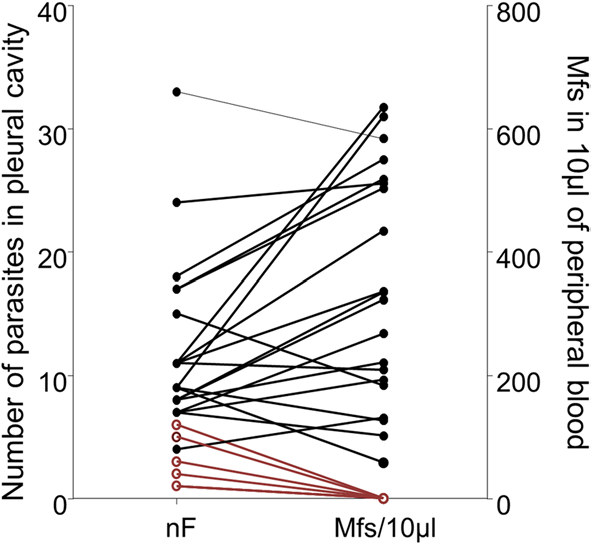
Microfilaremic gerbils have higher worm burden. Gerbils were infected with 40 L3 of *L. sigmodontis*. The adult filarial burden was analyzed at 70 days p.i. in the pleural cavity and the microfilaremia was quantified in 10 µl of peripheral blood. Number of filariae (nF) and microfilaremia are shown for each individual (*n* = 27). Microfilaremic gerbils (Mf^pos^) are in black (*n* = 20) and amicrofilaremic gerbils (Mf^neg^) are in red (*n* = 7). A solid line connect the filarial load and the microfilaremia for each gerbil. Correlation between the number of filariae and microfilaremia was analyzed by Spearmanʼs test: *r* = 0.81, *P* < 0.0001

Gerbils were inoculated subcutaneously with 40 L3. After 70 days of infection, all gerbils had adult filariae in their pleural cavity (8.8 ± 1.4 filariae per cavity) and 66% of them were microfilaremic (Mf^pos^) (Fig. 1). Microfilaremia was highly variable depending on the gerbils. Microfilaremic load was correlated to the adult filarial load (*r* = 0.81, *P* < 0.0001). In contrast with what was observed in BALB/c mice [24], amicrofilaremic gerbils (Mf^neg^) had significantly fewer parasites in the pleural cavity (Mf^neg^: n = 2.7 ± 0.78; Mf^pos^: n = 11.1 ±1.13; *t*_(24)_ = 4.32, *P* = 0.0002).

### Fibrotic polyps are present on the thoracic pleurae of infected gerbils

**Fig. 2:**
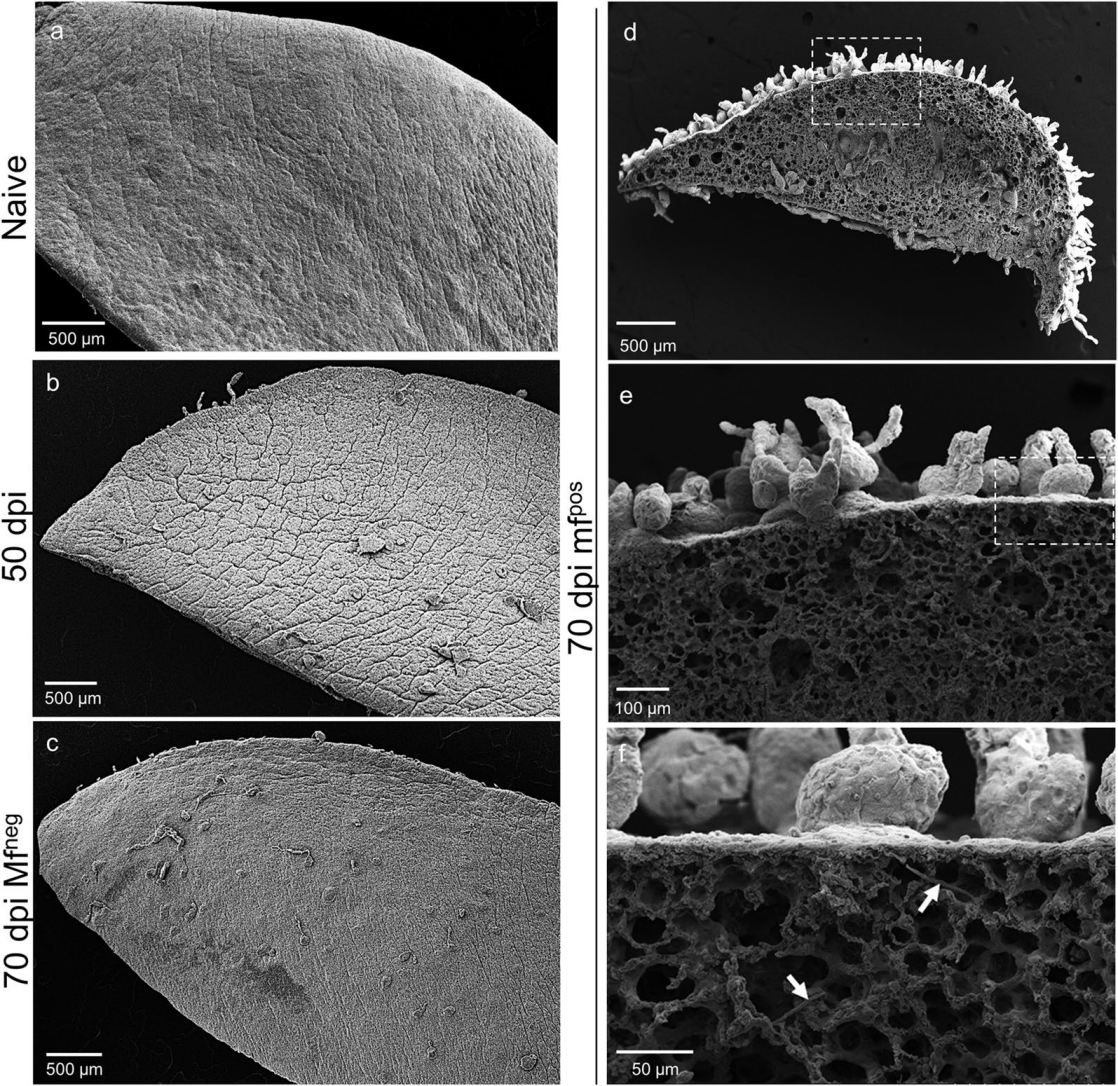
Microfilariae increase the formation of polypoid structures on the lungs. Lungs were recovered from naive (*n* = 3), 50 days (*n* = 3) and 70 days (=2 Mf^pos^ and 1 Mf^neg^) *L. sigmodontis* infected gerbils and prepared for scanning electron microscopy (SEM). **a** SEM micrograph of the lungs of an naive gerbil showing smooth lung visceral pleura. **b** SEM micrograph of the lung of 50 days infected gerbils and (**c**) 70 days amicrofilaremic (Mf^neg^) gerbils showing small and rare polyps on the visceral pleura. **d–f** SEM micrographs of the lungs of microfilaremic gerbils (Mf^pos^) showing lung visceral pleura with polypoid structures. **d** Overview. **e**, **f** Magnifications allowing microfilariae to be seen under the visceral pleura (white arrows)

**Fig. 3:**
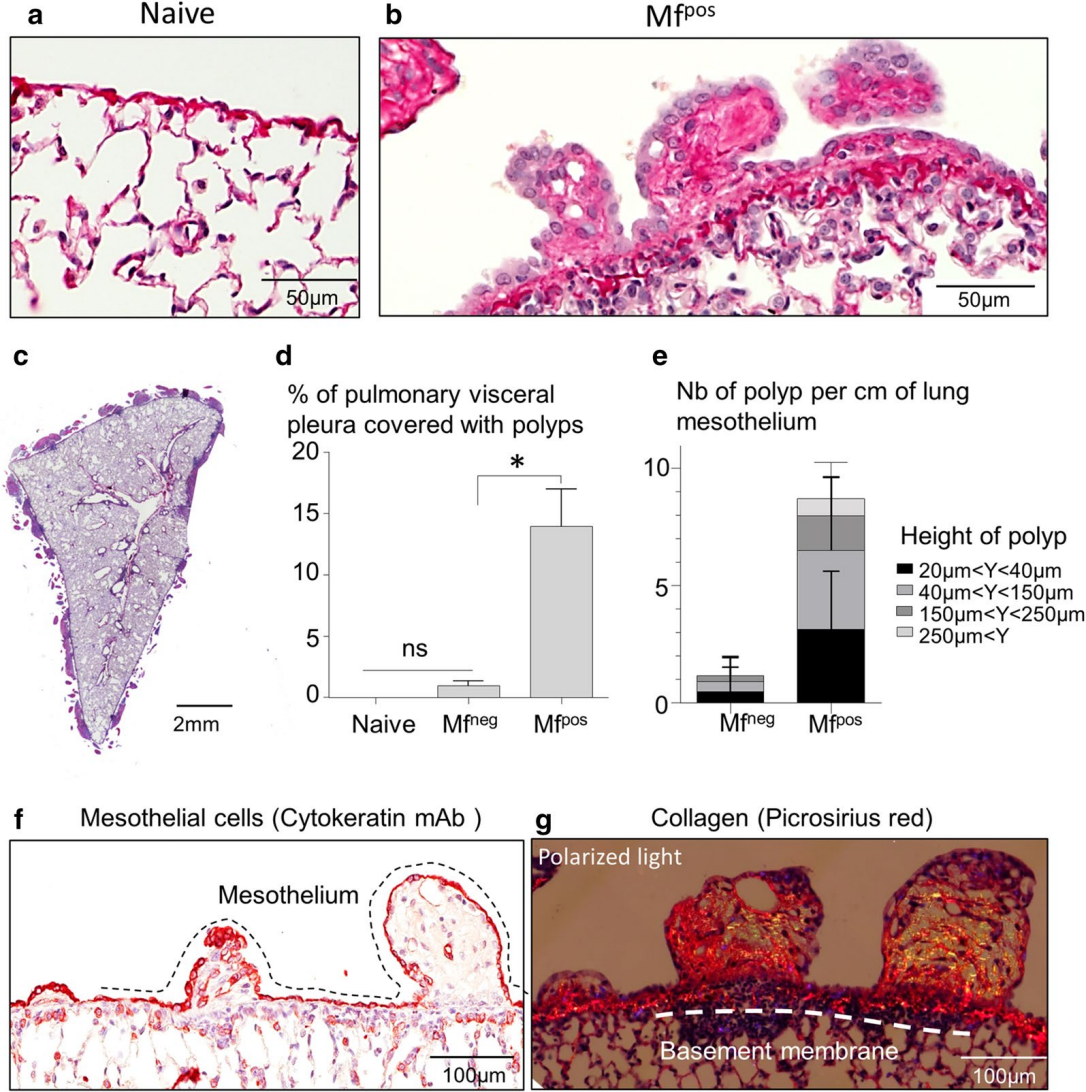
Lung polyps are rich in collagen and heterogenous in size. Lungs sections from naive and 70 days *L. sigmodontis-*infected gerbil (*n* = 19) were stained with hematoxylin & eosin. **a** Lungs of naive gerbil without pathology. **b** Polyps on the visceral pleura of Mf^pos^ gerbil; hyperplasic and hypertrophic mesothelium. **c** Left lung of a Mf^pos^ gerbil covered with polyps. **d** The extent of the pathology was quantified on the lungs of naive (*n* = 3), Mf^pos^ (*n* = 19) and Mf^neg^ gerbils (*n* = 7). The pathology is expressed as the proportion of the visceral pleura which is covered with polyps. Results are expressed as the mean ± SEM. Kruskal-Wallis followed by a Dunns multiple comparison tests: *P < 0.05 represent difference between naive and Mf^neg^ and Mf^pos^. **e** Polyps were characterized according to their height - small (20–40 µm), intermediate (40–150 µm) and large polyps (>150 µm) - and quantified (number of polyps/cm of lung mesothelium) in Mf^pos^ (*n* = 19) and Mf^neg^ (*n* = 7) gerbils. Results are expressed as the mean ± SEM. **f** Mesothelial cells (red) stained for cytokeratines. View of different development size of polyps. The dashed line shows the mesothelial layer. **g** Collagen fibers stained with picrosirius red. Polarized light observation of polyps reveals fibrosis. The dashed line shows the basement membrane

The lungs of naive gerbils presented a smooth visceral mesothelium (Figs. 2a and 3a). Whilst the lungs of 50-days-infected gerbils and Mf^neg^ gerbils displayed small and rare polyps on the visceral pleura (Fig. 2b, c), the visceral pleura of the lungs of Mf^pos^ gerbils showed a strong inflammatory reaction with the formation of polypoid structures (Figs. 2d–f and 3b–g). In addition, hypertrophy of mesothelial cells and hyperplasia of the mesothelium, similar to that described in BALB/c infected mice [24], were both observed on the lungs covered with polyps (Fig. 3b). Lung pathology was quantified as the percentage of the visceral pleura covered with polyps compared to the perimeter of both the left and right lungs (Fig. 3d). About 14% of the visceral mesothelium of Mf^pos^ lungs was covered with polyps, while only 1% of Mf^neg^ pleura showed these structures. Different sizes of polyps were observed on the visceral pleura of gerbils, from small to large polyps regarding the height of each polyp (Fig. 3e–g). Most of the polyps were relatively small (more than 70% measured between 20–150 µm in height). Larger polyps measuring more than 250 µm were rare (less than 1%). Polyps grew out from an area below the visceral mesothelium and remained surrounded by the mesothelial cells thoughout their development (cytokeratin staining; Fig. 3f). Polyps and hyperplastic mesothelium both contain a fibrous structure rich in collagen (yellow/orange bright fibers in polarized light; Fig. 3g). Polyps were also observed on the diaphragmatic pleura of all Mf^pos^ gerbils (Additional file 1: Figure S1 and Additional file 2: Fig.S2A,C-D) and occasionally on the pericardium of Mf^pos^ gerbils (Additional file 2: Figure S2b, e, f). Diaphragm pathology was quantified in the same way as for the lungs and about 14% of the parietal pleura of the diaphragm was covered with polyps in Mf^pos^ gerbils (Additional file 2: Figure S2g).

### Large polyps are vascularized

**Fig. 4:**
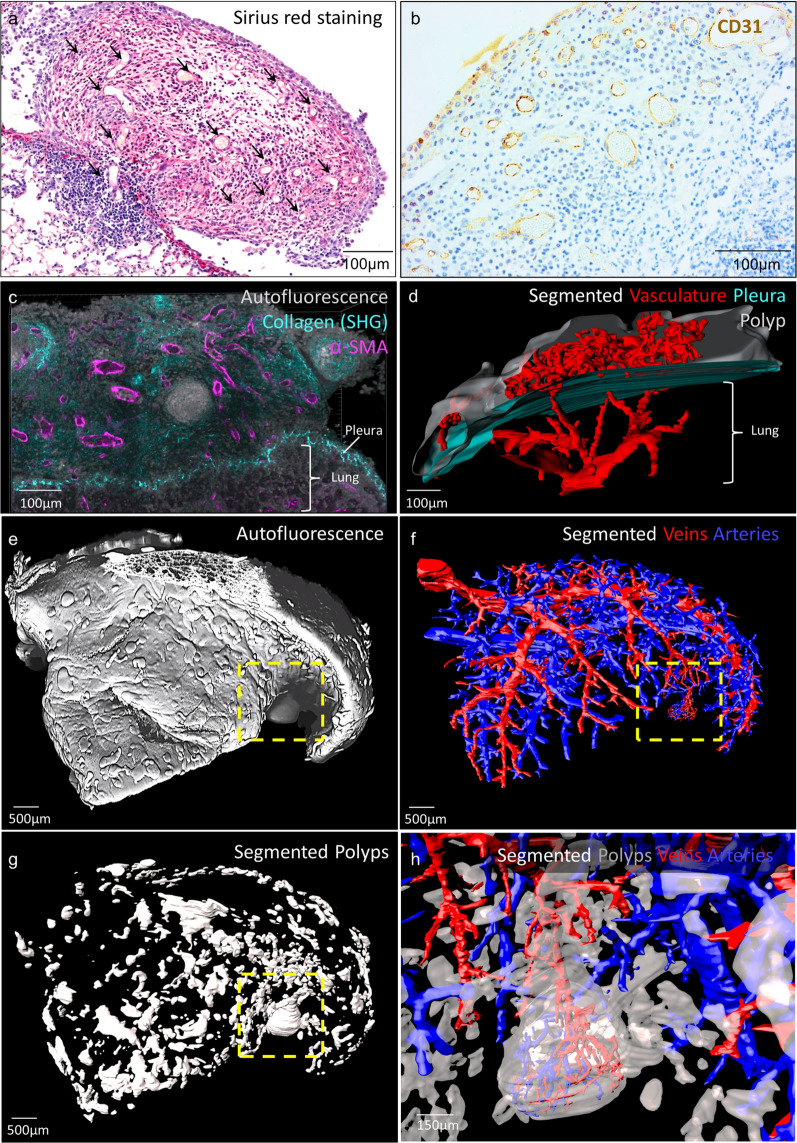
Large polyps are vascularized. Lungs from 70 days Mf^pos^ gerbils were paraffin-embedded. **a** Picrosirius red staining of a large polyp. Arrowheads indicate sections of blood vessels. Results are representative of *n* = 19 Mf^pos^ gerbils (**b**) CD31 staining of a large polyp (> 250 µm) showing a strong vascularization. **c–h** Lung tissue from 3 Mf^pos^ mice was processed for 3D imaging (see Additional file 4: Figure S4). **c**, **d** 500-µm-thick lung sections were imaged by 2-photon microscopy. **c** Blend mode vue (opaque colours) of a 131-µm z-stack showing αSMA expression (purple) by polyp’s blood vessels. Collagen is revealed by the Second Harmonic Generation (SHG) imaging (cyan) and tissue architecture by autofluorescence (white). **d** Segmentation of vasculature (red), polyp (gray) and pleura (cyan) in a 425-µm z-stack (step = 5 µm) showing pulmonary origin of polyp vasculature. **e–h** The post-caval lobe was imaged with a light sheet microscope and structures were segmented. **e** Blend mode vue (opaque colours) of tissue autofluorescence in a 3325-µm z-stack (step = 5 µm). **f** Segmentation of venous (red) and arterial (blue) trees. **g** Segmentation of polyps (gray). **h** Magnification of the framed area

Small polyps (< 150 µm) were never vascularized. Sections of blood vessels bordered by endothelial cells were only identified on polyps measuring more than 150 µm (Fig. 4a and Additional file 3: Fig. S3). They were all vascularized, as shown by anti-CD31 immunostaining and the presence of blood cells (Fig. 4b). To determine the origin of the vasculature in bigger polyps we took advantage of the development of tissue clearing methods to image large volumes of tissue in 3 dimensions [37, 39]. Briefly, lungs were recovered from paraffin blocks, stained for αSMA and cleared with Ethyl Cinnamate (see methods for more details). In the lung, αSMA is mainly expressed by smooth muscles surrounding bronchi, veins and arteries [40]. 2-photon imaging of thick tissue sections indicated that polyp vasculature also expressed αSMA (Fig. 4c). The segmentation of the vasculature showed that blood vessels in the polyps were connected to pulmonary blood circulation (Fig. 4 and Additional file 4: Movie S1) suggesting that a sprouting angiogenesis occurs in the polyps. The analysis of full lobes using a light sheet microscope confirmed the previous histological analysis (Additional file 5: Figure S4): no angiogenesis was observed in small polyps and only the larger ones presented vasculature (Fig. 4e–h and Additional file 6: Movie S2). These blood vessels originated from veins as well as arteries (Fig. 4h and Additional file 6: Movie S2). In addition, polyp vasculature was very dense, tortuous and disorganized (Fig. 4d, h, Additional file 4: Movie S1 and Additional file 6: Movie S2) which is somewhat similar to immature vasculature in some tumours [41, 42].

### Polyps of Mf^pos^ gerbils present massive cellular infiltrates

**Fig. 5:**
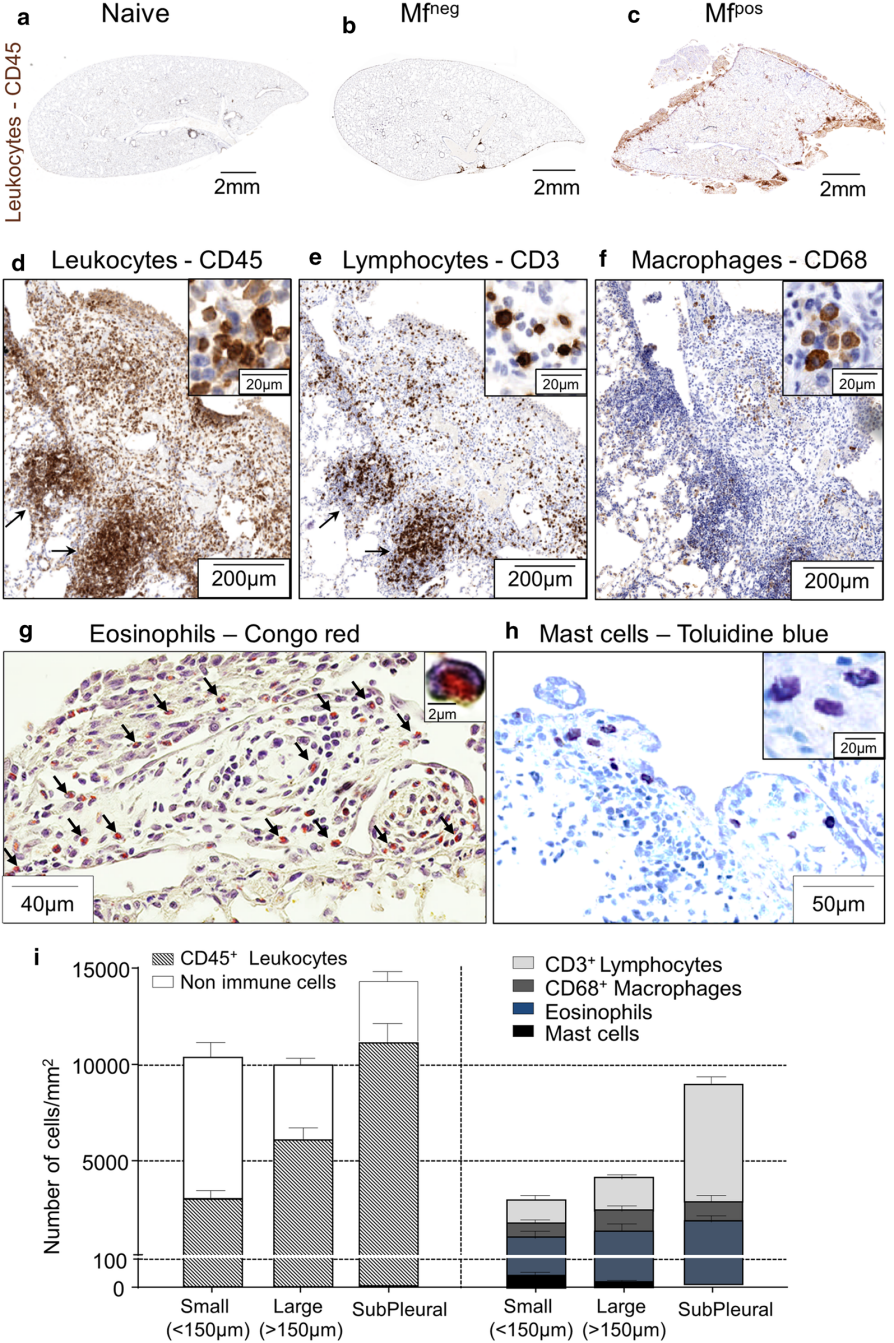
Polyps show a strong immune infiltration. Lungs were recovered from naive, Mf^neg^ and Mf^pos^ gerbils at 70 days p.i. Immune cells were stained in 4 µm thick sections. **a**–**f** Immunostainings were performed for leukocytes (CD45), lymphocytes (CD3) and macrophages (CD68). Overview of lungs section from naive (**a**), Mf^neg^ (**b**) and Mf^pos^ (**c**) gerbils stained with CD45 (brown). **d–f** View of a large polyp stained for CD45 (**d**), CD3 (**e**) and CD68 (**f**). Arrows show subpleural inflammatory foci. **g** Eosinophils were stained with Congo red; arrows indicate eosinophils. **h** Mast cells were stained with Toluidine blue. In all images, the right quadrant shows a zoom of positive cells. **i** Quantification of cell concentrations in small polyps (< 150 µm), large polyps (> 150 µm) and subpleural infiltrates. Results are expressed as the mean ± SEM (*n* = 7 Mf^pos^ gerbils for CD45, CD3, CD68 and toluidine blue staining and 4 for Congo Red)

Inflammation of the pleura was then analysed by histology (Fig. 5). The staining of the pan-leukocyte marker CD45 indicated that if Mf^neg^ animals showed a relatively mild pleural inflammation, the pleural area and the polyps of Mf^pos^ gerbils were infiltrated by numerous immune cells (Fig. 5a–c). Smaller polyps (< 150 µm) were composed of about 40% of leukocytes while in larger ones immune infiltrates reached 60% of total cells. Dense subpleural inflammatory foci mostly composed of immune cells (80%) were also observed under most large polyps and some small ones (Fig. 5c, d, i). Infiltrates in polyps and subpleural foci were mainly composed of CD3^+^ lymphocytes, CD68^+^ macrophages and eosinophils (Fig. 5e–g, i). A few mast cells could also be observed in polyps but they were absent from subpleural foci (Fig. 5h, i).

### Microfilaria and gravid females induces the formation of polyps on the visceral pleura

**Fig. 6:**
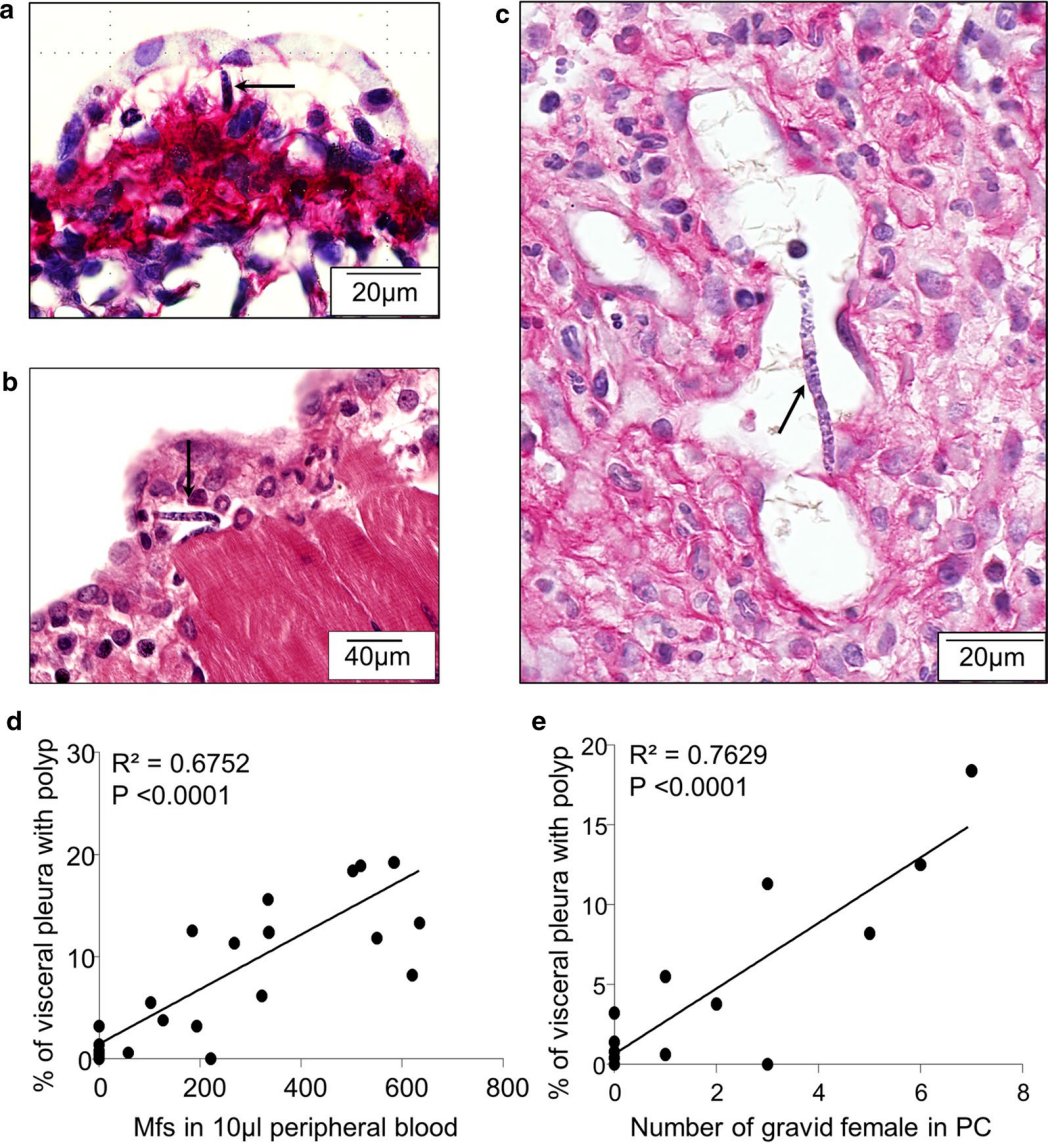
Microfilariae and gravid females induces the formation of polyps on the visceral pleura. Lungs from 70 days Mf^pos^ gerbils were recovered and polyps were analysed by histology. **a** Microfilaria (black arrow) in a small lung polyp. **b** Microfilaria (black arrow) in a small polyp of the diaphragm. Numerous eosinophils (lobed-shaped nucleus) near to microfilaria. **c** Microfilaria (black arrow) in a large lung polyp. **d** Linear regression between the pleural pathology and microfilaremia (Y (pleural pathology) = 0.02431 × X (microfilarial load) + 1.408; *R*^2^ = 0.67; *n* = 23 from 3 independent experiments). **e** Linear regression between the pleural pathology and gravid female (Y (pleural pathology) = 2.050 × X (number of gravid female) + 0.6341; *R*^2^ = 0.; *n* = 15 from 2 independent experiments)

Microfilariae were not only observed in the area below the polyps (Fig. 2f) but also in lung polyps (Fig. 6a, c) and in the inflamed diaphragmatic pleura (Fig. 6b). The relationship was linear between the number of Mf in peripheral blood and the pleural pathology (Spearmanʼs test; *n* = 21, *r* = 0 .79, *P* < 0.0001). A linear regression was used to study the strength and the direction of the relationship, showing that 67% (*R*^2^ = 0.6752, *P* < 0.0001) of the pleural pathology can be significantly explained by the microfilarial load (Pleural pathology = 0.2431 × microfilarial load + 1.408) (Fig. 6d). The observed pathology was related to the presence of adults (both males and females, males, females, or gravid females only); the relationship was always linear and positive (*n* = 26, *r* = 0.83, *P* < 0.001; *r* = 0.83, *P* < 0.001; *n* = 26, *r* = 0.68, *P* < 0.001; *n* = 14, *r* = 0.67, *P* < 0.01, respectively). However, the observed pathology was mainly driven by the presence of gravid females in the pleural cavity (gravid females: *R*^2^ = 0.76, *P* < 0.0001; both males and females : *R*^2^ = 0.6; *P* < 0.0001; males : *R*^2^ = 0.59, *P* < 0.0001; all females : *R*^2^ = 0.43, *P* < 0.0001). Thus, the linear regression showed that 76% (*R*^2^= 0.7629, *P* < 0.0001) of the pleural pathology can be significantly explained by the number of gravid female (Pleural pathology = 2.050 × number of gravid females + 0.6341) (Fig. 6e).

## Discussion

Here we show that infection of an immunocompetent and highly microfilaremic host with the filaria *Litomosoides sigmodontis* results in a strong pleural pulmonary manifestation with formation of vascularized eosinophilic polyps on the visceral pleura.

*Litomosoides sigmodontis* induces pleuro-pulmonary manifestations in a variety of rodents (cotton rats, rats, gerbils and BALB/c mice), which may differ in extent according to species, strain or individuals. However when we consider the pulmonary manifestations induced by *L. sigmodontis*, the available information mainly concerns the pleural space in which developing and adult filariae reside. Changes in lung tissue or in the pleura lining the thoracic cavity, the tissue with the closest contact with larvae and adult worms are less documented. The immune response in the thoracic cavity between the two pleurae has been extensively characterized at different stages of the filarial infection from the arrival of L3 larvae in the pleural space to the patent phase in susceptible (BALB/c) and resistant (C57BL/6) mouse models [15, 29, 31, 43–45]. Depending on the mouse strain, the mode of infection (natural through the vector or inoculation of a known number of L3) and the amount of the inoculum, the intensity of cell recruitment and the secretions of specific cytokines are different [25, 29, 46–48]. Regarding the mode of infection, subcutaneous (SC) injection of L3 may circumvent some of the skin immune responses and later pathological responses in the thoracic cavity. For example, the increased parasite load in IL-6 and NOD2-deficient mice, which show a delayed neutrophil recruitment at the site of infection, is overcome by subcutaneous infection [47, 49], resulting in a worm burden comparable to that of immunocompetent WT mice. However, mice deficient in S100A9 (which together with S100A8 forms a calprotectin that accounts for 40% of the cytosolic content of neutrophils) showed a similar decrease in parasite burden and an identical cell response in the pleural cavity, whether subcutaneously inoculated or naturally infected [50]. Also, at 8 days p.i., i.e. when the L3 settled in the pleural cavity, the recovery rate was similar in naturally or subcutaneously infected mice [15]. In gerbils, the presence of polyps on the visceral pleura was described in both animals naturally infected [35] or inoculated with *L. sigmodontis.* However, as it is not possible to control the inoculum, natural infections can lead to very variable parasite burden. The previous reports of pulmonary pathology after natural infection presented much higher worm burdens than those obtained after SC injection of 40 L3 larvae (depending on studies, an average of 20–250 adult parasites was isolated from the thoracic cavity *vs* 8–10 after SC infection) [19, 21, 24, 33, 35]. These massive parasite loads could explain an earlier onset of the pathology in some studies and hide the microfilaria-specific effect observed here. This suggests that if circumventing natural immune responses in the skin does not necessarily alter the pleuropulmonary phenotype, worm burden could have an impact. Indeed, until now, the pleuropulmonary manifestations have been considered to be caused mainly by adult worm products [16, 21, 35]; they were not seen to be associated with the presence of microfilariae. To support this proposition, some lung pathologies were observed even before the patent phase, when adult worms are present but the microfilariae are not yet released [20, 21]. In our experimental system, small and rare polyps were observed on the pleura of 50-days-infected gerbils, i.e. before the production of microfilariae but only microfilaremic gerbils have numerous and large polyps on the visceral pleura. Thus it is likely that developing filariae present in the pleura cavity are responsible for the initiation of the pleuropulmonary pathology but the release of microfilariae can further increase the phenomenon. Indeed, microfilariae are present in small polypoid structures and the number of microfilariae present in the peripheral blood is correlated with the extent of the pathology. Moreover, the formation of polyps could be enhanced by inflammation of the mesothelium in contact with the microfilariae as pleural mesothelium reacts rapidly to infection by producing cytokines which recruit cells [51]. Regarding the mouse strain, a strong proliferation of resident macrophages is observed in the pleural cavity of C57BL/6 mice whereas the increase in macrophage population is mainly due to an influx of circulating monocytes in BALB/c mice [45]. The mesothelial cells contiguous to the visceral pleura have been shown to produce inflammatory chemokines such as CCL2 or CXCL12 in both strains of mice [29, 52]. Pathological alterations in the lung caused by L3 larvae migration were noted with hemorrhages and granulomas observed in the pulmonary parenchyma of BALB/c mice [15]. Later the presence of adult filariae in the pleural space leads to pleural hypertrophy, and in the presence of microfilariae, pleural hyperplasia with dense mesh of collagen fibers (a signature of fibrosis) was also described [24]. Pathological changes of the visceral mesothelium was also described in highly microfilaremic and immunocompetent hosts of *L. sigmodontis*. The proliferative reaction of the visceral mesothelium lead to the formation of polyps that protrude from the surface of the pleura. Polyps are also present in other areas of the thoracic cavity such as the pericardium and diaphragmatic pleura. Such pathologies were noticed in the gerbil *M. unguiculatus* [19] or in albino rats [53] and to a much lesser extent in the cotton rats [35] or in *Mastomys natalensis* [54] but never in the mouse [24].

Similar to BALB.Xid mice that are deficient in B1 lymphocytes, gerbils present an deficiency of antibody response to T-independent antigen [55]. BALB.Xid mice show higher susceptibility to *L. sigmodontis* infection resulting in higher microfilarial burdens which was accompanied by a lower Th2 response and the inability of BALB.Xid B cells to produce IL-10 [56]. An alteration in the Th2 response could explain the strong permissiveness of the gerbil to infection with *L. sigmodontis*. However, the immune response is still Th2-oriented because a strong eosinophilic response in the polyps is observed. Other cells can then stimulate the Th2 response, such as innate lymphoid type 2 cells, which have been shown to play a role in triggering the Th2 response to helminth infection (including filariasis) by secreting specific cytokines [57, 58]. This response plays an important role in the observed pathology because in the absence of eosinophils, the pleura of mice does not show any signs of hyperplasia and therefore protects against fibrosis [24]. Although eosinophils are known to be a central feature of the host response for the elimination of adult worms and microfilariae [59–61], they also accelerate the development of the parasite in the early stages of infection [44] and participate in the damages in the event of hyperresponsiveness [62–64]. In lymphatic filariasis, eosinophils have a dual role regarding their involvement in the pathogenesis associated with the tropical pulmonary eosinophilia. On one hand they participate in the destruction of the microfilariae trapped in the pulmonary microcirculation but on the other hand they are responsible for some of the damage to the lungs. This happens through the release of the components of the eosinophilic granule, such as major basic protein, eosinophil-derived neurotoxin and eosinophil cationic protein or mediators, such as transforming growth factor, which act directly on target fibroblasts to promote fibrosis [3, 62, 64]. The host must then regulate its eosinophilic balance to resolve the infection and avoid tissue damage such as polyps on the pleura. Mast cells are also involved in the immune response to infection by helminths including filaria [47, 65–67] but appears to have a limited role during the infection with *L. sigmodontis* [68]. As only a very few mast cells were observed in polyps, it seems unlikely that they play an important role in the observed pathology.

The etiology of the polyps is currently unknown. They are likely the result of a proliferative reaction of the visceral mesothelium. Initially they appeared as a slight uplift of the mesothelium, then as they develop they are invaded by inflammatory cells such as eosinophils, macrophages, lymphocytes and a few mast cells. Larger polyps formed in less than 20 days. The growth of polyps was associated with the rapid development of imperfect vascularization. This is reminiscent of the tortuous and leaky vessels observed in some tumors [41]. However, it remains to be elucidated whether the vascularization here is associated with the explosive inflammatory response of the host, the hypoxic environment in the polyps and/or the parasite itself, through the release of angiogenic factors [69–71]. Little information on direct pro-angiogenic functions of filarial antigens is available. For example Ov-ASP proteins from *Onchocerca volvulus* may directly induce an angiogenic response [72, 73] and may therefore contribute to corneal neovascularization in onchocercal keratitis. After the formation of subcutaneous nodules around adult *O. volvulus* worms, angiogenesis and lymphangiogenesis occurs inside associated with angio-lymphangiogenic factors such as CXCL12 and VEGF-C [74]. Asparaginyl-tRNA synthetase (*Bm*AsnRS) of *Brugia malayi* induced *in vitro* proliferation and tube formation by endothelial cells but also vasodilatation and lengthening of vessels *ex vivo* [75]. In addition, *Dirofilaria immitis* has an indirect effect on angiogenesis by stimulating the secretion of the pro-angiogenic factor vascular endothelial growth factor A (VEGF-A) by the endothelial cells [76]. The presence of the filariae/microfilariae may stimulate the formation of new vessels which are usually beneficial defense processes generated in response to parasitic infection. Indeed the formation of new vessels results in a local influx of plasma and inflammatory cells to encounter the parasite [77, 78]. These inflammatory cells in turn can help amplify the angiogenic signal. The role of eosinophils in tissue remodeling and angiogenesis in allergic disease is also well documented [79–82]. They can release several proangiogenic mediators such as VEGF or eosinophil peroxidase [79, 83, 84] and can therefore contribute in the neovascularization process. Macrophages exhibit a variety of cellular functions including the secretion of growth factors, enzymes and chemokines, which are potentially important for the vascular growth and remodeling during angiogenesis and lymphangiogenesis [85]. Thus, all these cells can act in synergy to promote the formation of new vascularization.

## Conclusions

This study shows that although the pathology is initiated by the presence of young adult filariae in the pleural cavity of gerbils, the presence of gravid females and then microfilariae exacerbates the inflammation of the pleural mesothelium. Whatever the rodent host, the pathological changes in the visceral pleura seem to follow the same etiology: the mesothelial cells become hypertrophic before the release of microfilariae. However, each rodent species has its own specificities. For example, unlike cotton rats, white rats and gerbils, no polyp has ever been observed in mice regardless of their permissiveness to infection and immune status. When the infection becomes patent with the appearance of microfilariae in the pleural cavity and then in the bloodstream, the damage already initiated by the presence of adult worms was exacerbated in high microfilaremic hosts. For example, only microfilaremic mice have a hyperplasic and fibrous visceral pleura. In amicrofilaremic gerbils, the polyps are punctual and remain small whereas they can become very large and vascularized in microfilaremic gerbils.

## Supplementary information

**Additional file 1: Figure S1.** Simplified anatomical diagram of the thoracic.

**Additional file 2: Figure S2.** Polyps are present on diaphragm and heart of microfilaremic gerbils.

**Additional file 3: Figure S3.**Vascularisation of polyps measuring between 150–250 µm.

**Additional file 4: Movie S1.** Analysis of polyp vasculature by 2-photon microscopy. Lungs were recovered from 70 days naive and microfilaremic gerbils and paraffin-embedded. After histological analysis, the left lung lung tissue was recovered from paraffin blocks, sliced with a vibratome (500 μm), stained for αSMA and processed for 2-photon 3D imaging. The movie first shows (up to 00:06) the 3D view of autofluorescence (white) and αSMA (purple) signals in blend mode vue (opaque colours) followed by the view of manually segmented vasculature (red), pleura (cyan) and polyps (white).

**Additional file 5: Figure S4.** Analysis of polyps by light sheet microscopy.

**Additional file 6: Movie S2.** Analysis of polyp vasculature by light sheet microscopy. Lungs were recovered from 70 days naive and microfilaremic gerbils and paraffin-embedded. After histological analysis, the post-caval lobe of the lung was recovered from paraffin blocks, stained for αSMA and processed for full lobe Light Sheet 3D imaging. The movie first shows (up to 00:13) the 3D view of autofluorescence (white) signal in blend mode vue (opaque colours) followed by the view of manually segmented veins (red), arteries (blue) and polyps (white).

## Data Availability

All data supporting the findings of this article are included in the article and its additional files.

## Abbreviations

αSMA: Alpha-smooth muscle actin
BSA: Bovine serum albumin
HMDS: Hexamethyldisilane
IL: Interleukin
L3: Infective third-stage larvae
Mf^neg^: Amicrofilaremic
Mf^pos^: Microfilaremic
NGS: Neutral goat serum
PBS: Phosphate-buffered saline
p.i.: Post-inoculation
SEM: Scanning electron microscopy
