# Supplementary figures and images for: Microfilaria-dependent thoracic pathology associated with eosinophilic and fibrotic polyps in filaria-infected rodents
Source: Parasit Vectors. 2020 Nov 7;13:551. doi: 10.1186/s13071-020-04428-0 (PMC7648300; doi:10.1186/s13071-020-04428-0)

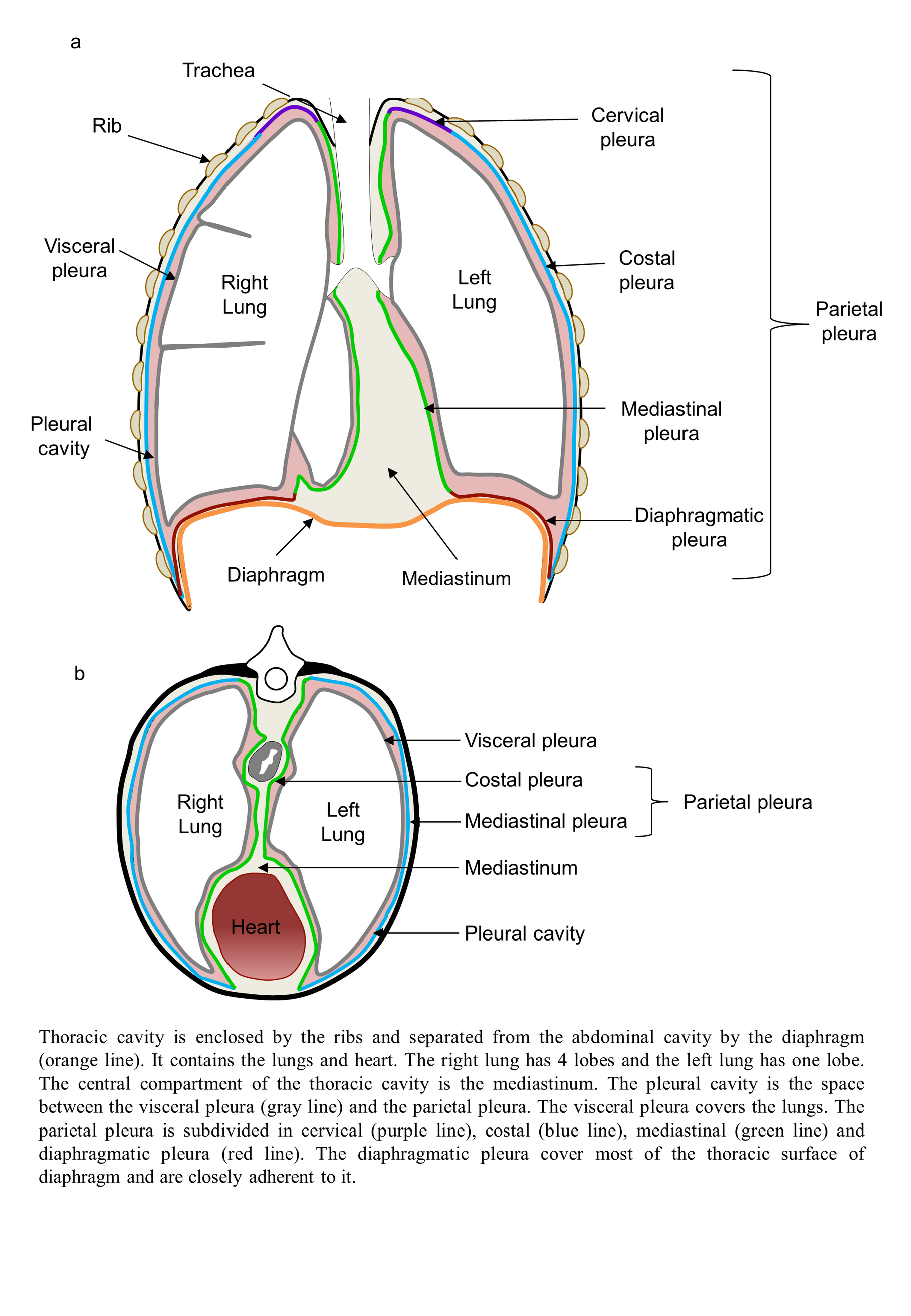

Supplement: Supplementary file 1 — Additional file 1: Figure S1. Simplified anatomical diagram of the thoracic. [file 13071_2020_4428_MOESM1_ESM.tif]

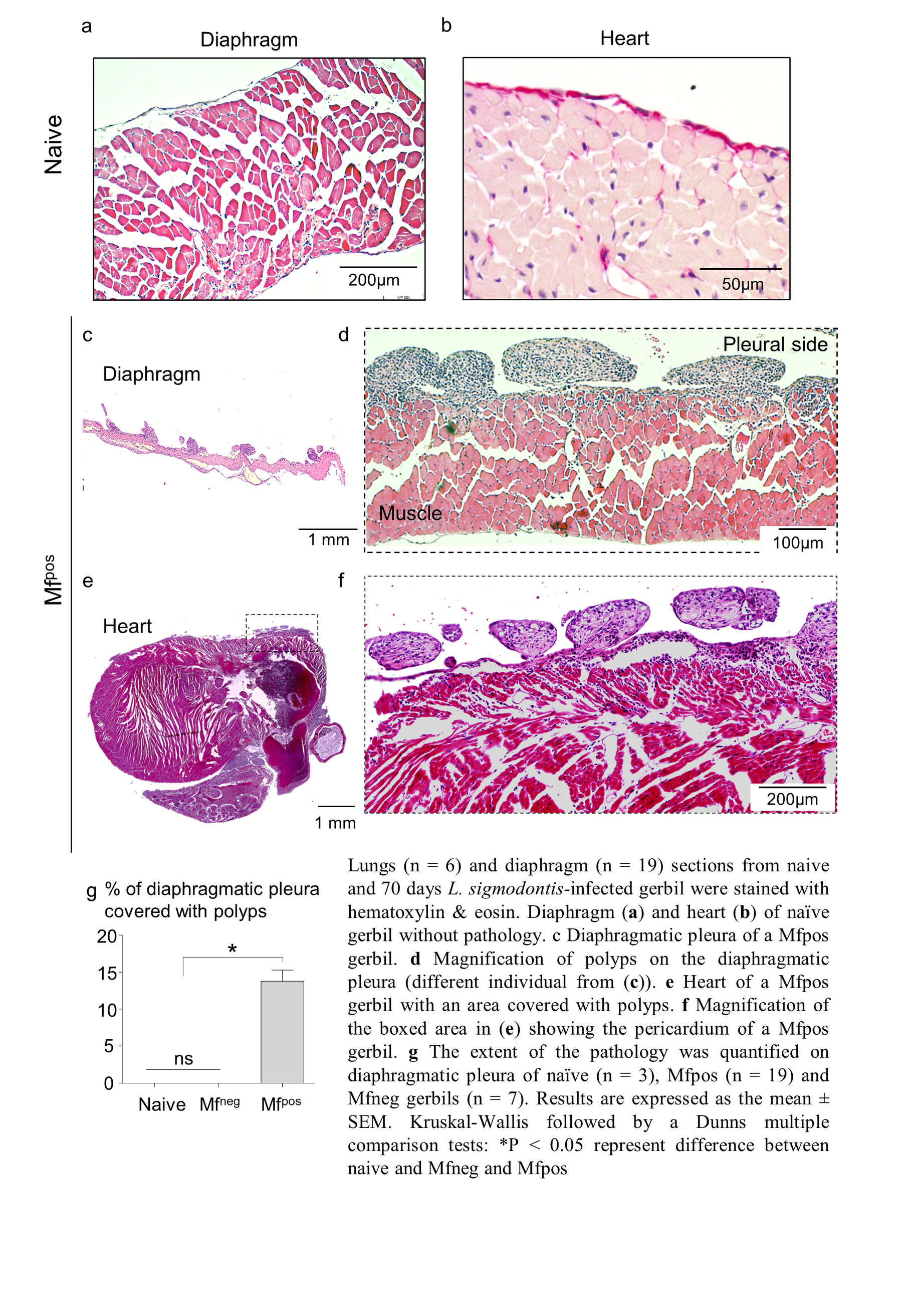

Supplement: Supplementary file 2 — Additional file 2: Figure S2. Polyps are present on diaphragm and heart of microfilaremic gerbils. [file 13071_2020_4428_MOESM2_ESM.tif]

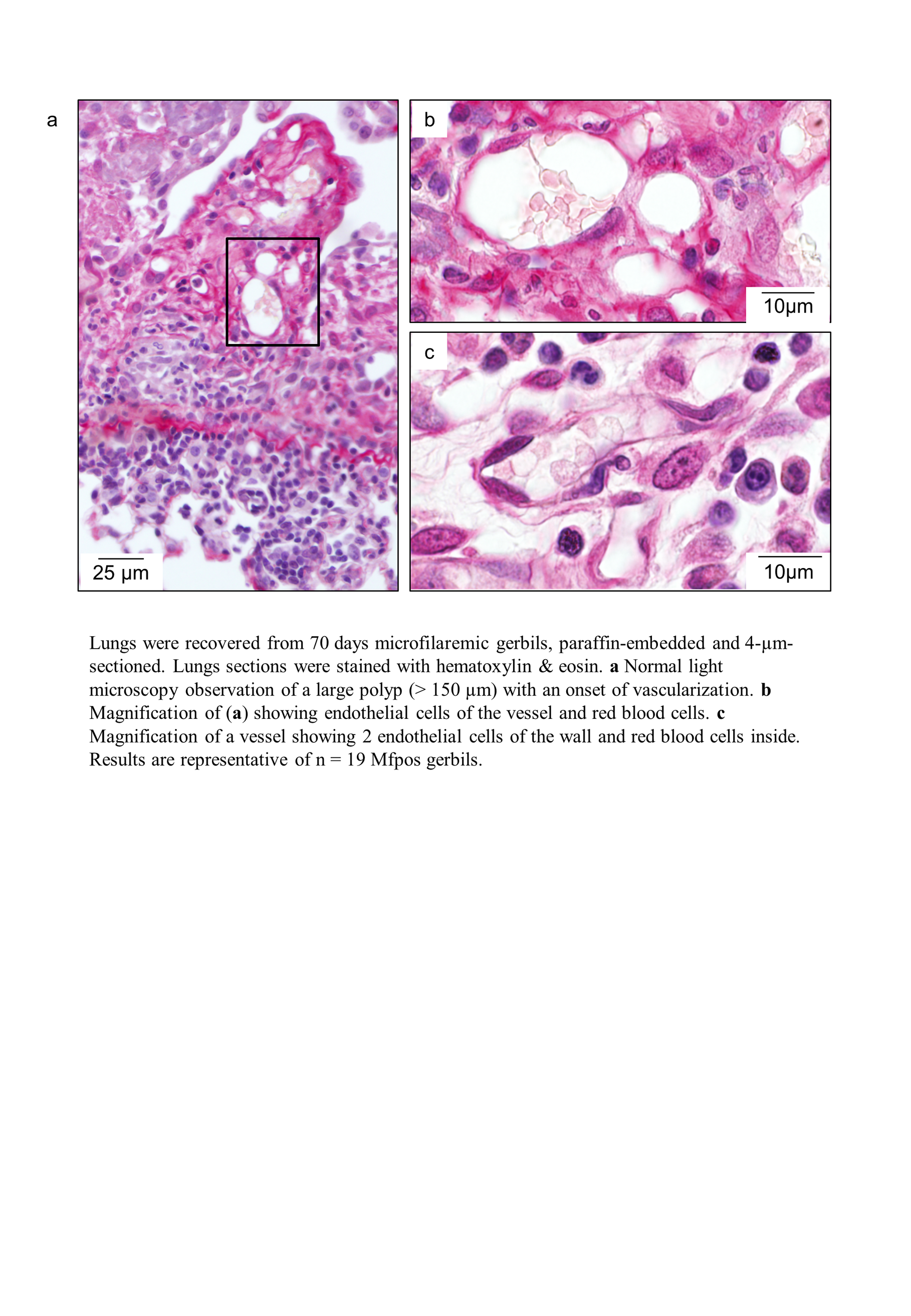

Supplement: Supplementary file 3 — Additional file 3: Figure S3.Vascularisation of polyps measuring between 150–250 µm. [file 13071_2020_4428_MOESM3_ESM.tif]

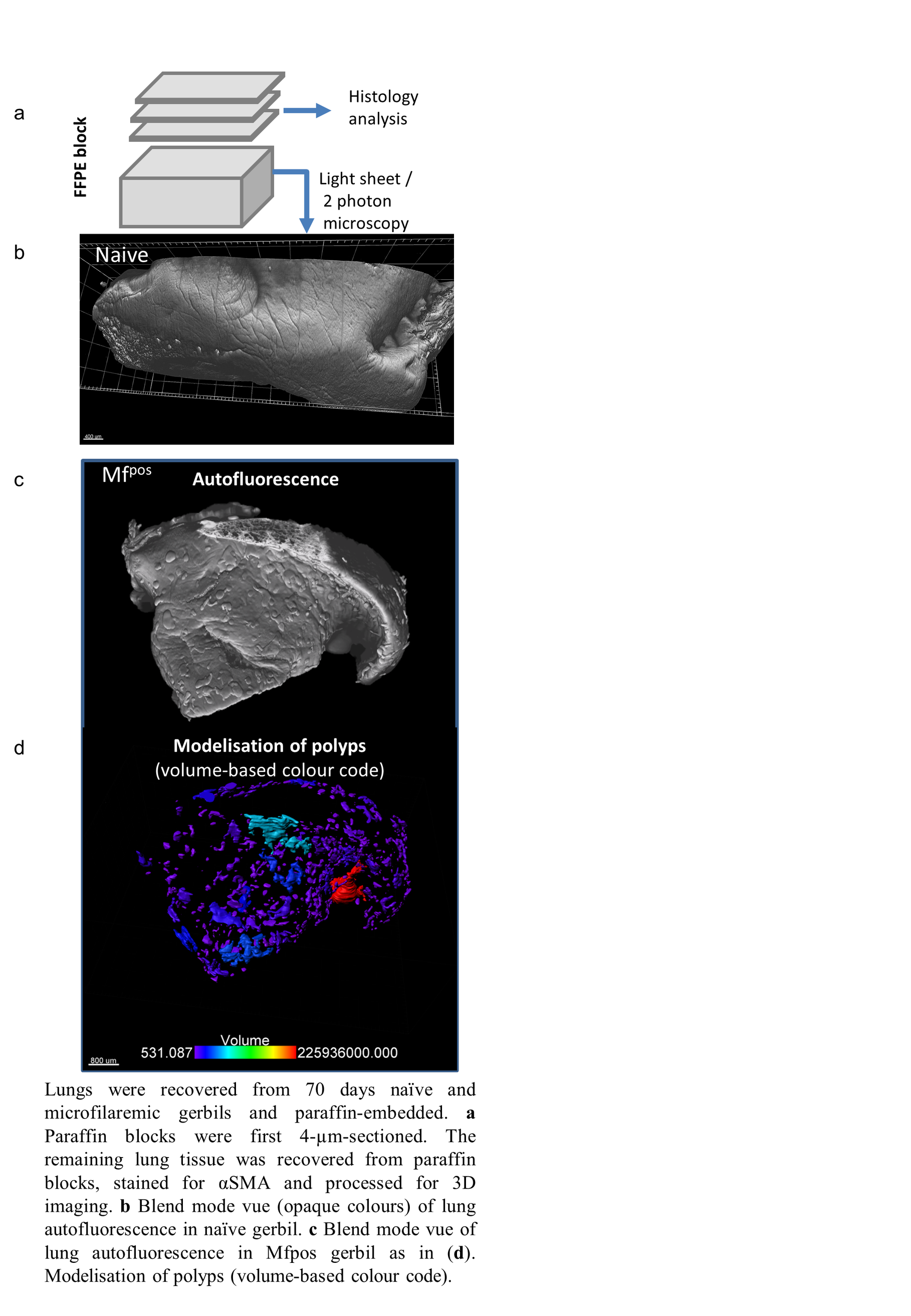

Supplement: Supplementary file 5 — Additional file 5: Figure S4. Analysis of polyps by light sheet microscopy. [file 13071_2020_4428_MOESM5_ESM.tif]
